# Periostin Contributes to the Acquisition of Multipotent Stem Cell-Like Properties in Human Mammary Epithelial Cells and Breast Cancer Cells

**DOI:** 10.1371/journal.pone.0072962

**Published:** 2013-08-29

**Authors:** Xiaowei Wang, Jia Liu, Zhe Wang, Yangmei Huang, Weiping Liu, Xiao Zhu, Yao Cai, Xiaoguang Fang, Shuyong Lin, Li Yuan, Gaoliang Ouyang

**Affiliations:** 1 State Key Laboratory of Cellular Stress Biology, School of Life Sciences, Xiamen University, Xiamen, China; 2 Laboratory of Stem Cells and Tumor Metastasis, School of Life Sciences, Xiamen University, Xiamen, China; 3 Department of Stem Cell Biology and Regenerative Medicine, Lerner Research Institute, Cleveland Clinic, Cleveland, Ohio, United States of America; Fox Chase Cancer Center, United States of America

## Abstract

Periostin (POSTN), a recently characterised matricellular protein, is frequently dysregulated in various malignant cancers and promotes tumor metastatic growth. POSTN plays a critical role in the crosstalk between murine breast cancer stem cells (CSCs) and their niche to permit metastatic colonization. However, whether pro-metastatic capability of POSTN is associated with multipotent potentials of mesenchymal stem cells (MSCs) has not been documented. Here we demonstrate that POSTN promotes a stem cell-like trait and a mesenchymal phenotype in human mammary epithelial cells and breast cancer cells. Interestingly, ectopic overexpression of POSTN or recombinant POSTN treatment can induce human mammary epithelial cells and breast cancer cells differentiation into multiple cell lineages that recapitulate part of the multilineage differentiation potentials of MSCs. Moreover, POSTN is highly expressed in bone marrow-derived MSCs and their derived adipocytes, chondrocytes, and osteoblasts *in vitro*. Furthermore, POSTN promotes the growth of xenograft tumors *in vivo*. POSTN-overexpressing human mammary epithelial cells enhance breast tumor growth and metastasis. These data thus provide evidence of a new role for POSTN in mammary epithelial neoplasia and metastasis, suggesting that epithelial cancer cells might acquire CSC-like traits and a mesenchymal phenotype, as well as the multipotent potentials of MSCs to promote tumorigenesis and metastasis. Therefore, targeting POSTN and other extracellular matrix components of tumor microenvironment may help to develop new therapeutical strategies to inhibit tumor metastasis.

## Introduction

Tumor development is a continuous reciprocal interaction between cancer cells and their surrounding microenvironment, in which stromal cells and the extracellular matrix (ECM) play a decisive role in tumorigenesis and metastasis [1], [2]. The ECM composition of tumor stroma is characteristically modified, in part by cancer cells, which secrete their own ECM proteins and use the ECM proteins secreted by neighboring stromal cells to create a specialized supportive microenvironment for the initiation and growth of the primary tumor and for tumor metastatic dissemination to distant organs.

Matricellular proteins are a group of nonstructural ECM proteins that includes osteonectin (SPARC), CCNs, tenascins, thrombospondins, osteopontin (OPN), periostin (POSTN), and βig-h3 (TGFBI). As a matricellular protein, POSTN, also called osteoblast-specific factor 2 (OSF-2), was originally isolated from MC3T3-E1 osteoblast cells as an important regulator of bone and tooth formation and maintenance [3], [4]. POSTN is also involved in cardiac valve development and healing [5], [6], [7], [8]. POSTN is often associated with the need for ECM remodeling and cell migration during embryonic development and is also highly expressed at sites of injury or inflammation within the adult organism, and thus plays a critical role in embryonic development, tissue injury, inflammation, and fibrosis [9], [10], [11], [12], [13], [14], [15], [16]. As a mesenchyme-specific gene product, POSTN is frequently overexpressed in a wide variety of human tumors [14], [17], [18], [19]. POSTN potently promotes the metastatic development of colon cancer by both preventing stress-induced apoptosis in the cancer cells and augmenting endothelial cell survival to promote angiogenesis via activating the Akt pathway [17]. POSTN also plays a role in human breast cancer progression by inducing angiogenesis [20]. Increased POSTN serum levels are found in human breast cancer patients with bone metastases [21], [22]. A recent report demonstrated that stromal POSTN is a key limiting factor that regulates the lung metastasis of mouse breast tumors and that POSTN can augment Wnt signalling in mouse breast cancer stem cells (CSCs) [23]. These studies suggest that POSTN plays an important role in breast tumor progression. However, whether pro-metastatic capability of POSTN is associated with multipotent potentials of mesenchymal stem cells (MSCs) is still unknown [24].

To further understand the roles of POSTN in breast cancer progression, we characterised the effects of POSTN on the stemness and multilineage differentiation potentials and tumorigenicity of human mammary epithelial cells and breast cancer cells (BCCs). Results show that overexpression of POSTN and POSTN treatment promote a stem cell-like and mesenchymal phenotype in human mammary epithelial cells and BCCs. Intriguingly, the POSTN-overexpressing cells undergo MSC-like multilineage differentiation and render cells more tumorigenic and metastatic. These findings highlight the relevance of POSTN in breast tumor growth and metastasis as an extracellular matrix component of tumor microenvironment via promoting CSC-like traits and MSC-like phenotypes.

## Results

### POSTN Promotes a Stem Cell-like Phenotype in Human Mammary Epithelial Cells and BCCs

**Figure 1:**
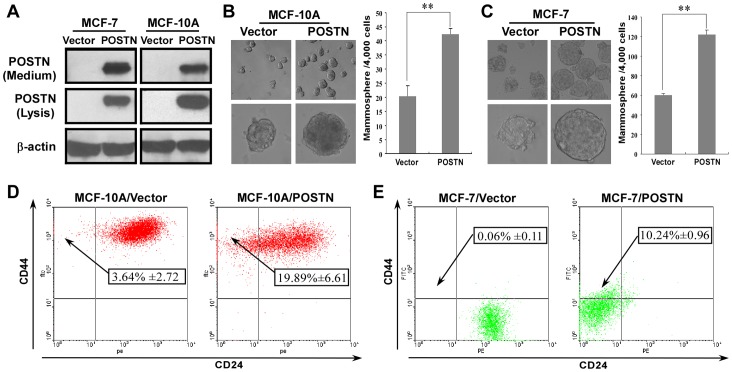
Human mammary epithelial cells and BCCs with ectopic expression of POSTN exhibit stem-like cell properties. **A.** Generation of stable cell lines that constitutively expressed either POSTN or empty vectors. **B, C.** Mammosphere formation from MCF-10A/Vector, MCF-10A/POSTN, MCF-7/Vector, and MCF-7/POSTN cells. The data are the means ± SD. **P<0.01. **D, E.** The frequency of CD44^high^/CD24^low^ stem-like cells in MCF-10A/Vector, MCF-10A/POSTN, MCF-7/Vector, and MCF-7/POSTN cells. The percentages of CD44^high^/CD24^low^ cells are shown as means ± SD.

To further demonstrate the roles of POSTN in human breast cancer progression, we first used an immortalized, non-transformed human mammary epithelial cell line (MCF-10A) and a low-tumorigenic human breast cancer cell line (MCF-7) to generate stable cell lines that constitutively expressed either POSTN or empty vectors by retroviral infection (Figure 1A). We used these cell lines to test whether POSTN promotes a stem cell-like phenotype in human mammary epithelial cells and BCCs. Mammosphere formation assays revealed a significant increase in the size and number of mammospheres in POSTN-expressing MCF-10A and MCF-7 cells compared with their vector-transduced cells (Figure 1B, C). POSTN-specific antibody treatment significantly decreased the number of mammospheres in MCF-10A/POSTN and MCF-7/POSTN cells (data not shown). These results demonstrated that POSTN promotes mammosphere formation. Moreover, the numbers of the CD44^high^/CD24^low^ stem cell-like subpopulation were significantly increased, from 3.64% in the MCF-10A/Vector control to 19.89% in MCF-10A/POSTN cells (P<0.01) (Figure 1D), and from 0.06% in MCF-7/Vector cells to 10.24% in MCF-7/POSTN cells (P<0.01) (Figure 1E). Furthermore, we sorted out the main non-stem cell subpopulation in MCF-10A and MCF-7 cells and then overexpressed POSTN in these non-stem cell subpopulation. We found that the percentages of CD44^high^/CD24^low^ subpopulation in the POSTN-overexpressing non-stem MCF-10A and MCF-7 cells were increased to 14.67% and 9.09%, respectively (P<0.01) (Figure S1). Together, these results indicate that POSTN promotes a stem cell-like phenotype in human mammary epithelial cells and BCCs.

### Overexpression of POSTN Induces a Mesenchymal Phenotype in Human Mammary Epithelial Cells and BCCs

**Figure 2:**
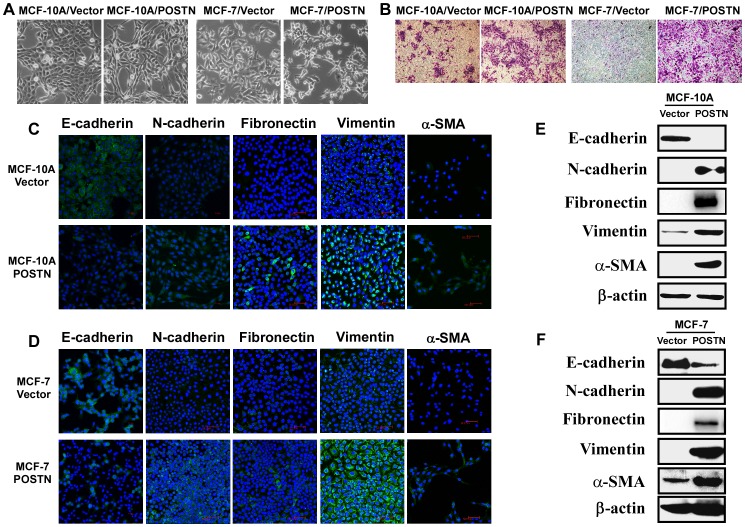
POSTN promotes a mesenchymal phenotype in MCF-10A and MCF-7 cells. **A.** POSTN-overexpressing cells exhibit a mesenchymal-like morphology. **B.** POSTN promotes cell invasion of human mammary epithelial cells and BCCs as detected by a matrigel-coated transwell invasion assay. **C, D.** Immunofluorescence analysis revealed that the mesenchymal markers N-cadherin, fibronectin, vimentin and α-SMA in POSTN-expressing cells were increased while the epithelial marker E-cadherin was decreased. **E, F.** POSTN-expressing cells show increased levels of N-cadherin, fibrnectin, vimentin and α-SMA and decreased E-cadherin. Expression of epithelial and mesenchymal markers was analysed by western blotting.

Recently, stem cell characteristics have been linked to the EMT program [25]. As shown in Figure 2A, compared with the vector-infected cells, POSTN-expressing MCF-10A and MCF-7 cells underwent a morphological change from a cobblestone-like epithelial morphology to an elongated fibroblast-like morphology. Enhanced cell invasion is a hallmark of EMT phenotype acquired by epithelial cells. We thus performed *in vitro* Matrigel transwell invasion assay to determine the effects of POSTN on cell invasion of MCF-10A and MCF-7 cells. Overexpression of POSTN results in a clear and potent invasive phenotype in both human mammary epithelial cells and BCCs *in vitro* (Figure 2B). Immunofluorescence analysis showed that the mesenchymal markers N-cadherin, fibronectin, vimentin and α-SMA in POSTN-expressing cells were increased while the epithelial marker E-cadherin was decreased (Figure 2C, D). Western blotting analysis further confirmed that ectopic overexpression of POSTN resulted in down-regulation of epithelial marker E-cadherin and up-regulation of mesenchymal markers N-cadherin, fibronectin, vimentin and α-SMA in human mammary epithelial cells and BCCs (Figure 2E, F). These data indicate that POSTN promotes a mesenchymal phenotype in human mammary epithelial cells and BCCs.

### POSTN Augments Multilineage Differentiation Potentials of Human Mammary Epithelial Cells and BCCs

**Figure 3:**
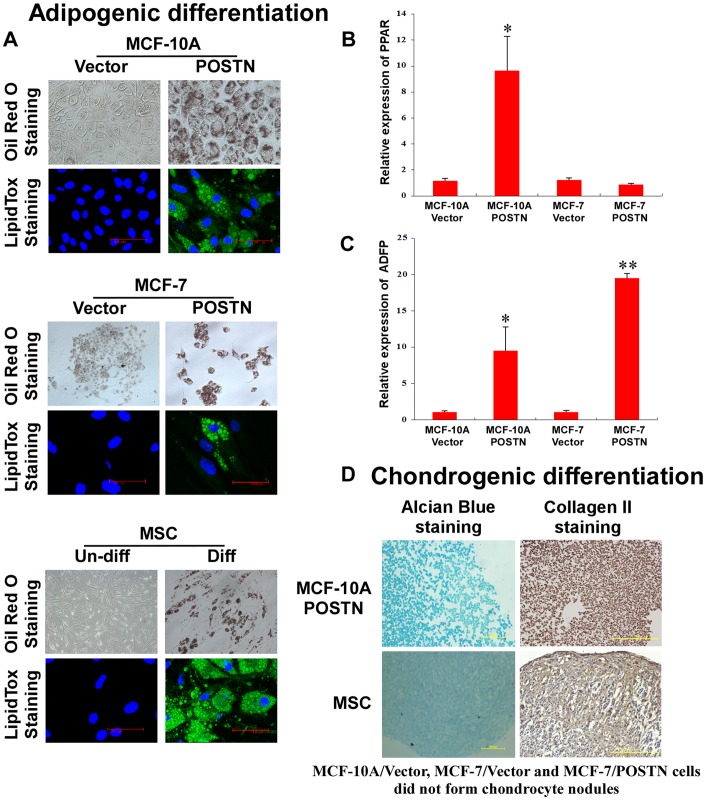
POSTN induces adipogenic and chondrogenic differentiation. **A.** Following adipogenic differentiation, MCF-10A/POSTN, MCF-7/POSTN cells and hMSCs stained positive with oil red O (top) and fluorescent LipidTox, which stains oil droplets (bottom). **B, C.** Real-time RT-PCR analysis for the expression of the adipocyte markers *PPARγ*and *ADFP* in MCF-10A and MCF-7 cells and their POSTN-overexpressing cells subjected to adipocyte differentiation for 21 days. The data are means ± SD. *P<0.05, **P<0.01. **D.** Chondrocytic nodules formed by MCF-10A/POSTN cells and hMSCs stained positive with alcian blue 8 GX (left panel). Immunohistochemistry was performed on chondrocyte sections using antibody against collagen II (right panel). MCF-10A/Vector cells, MCF-7/Vector and MCF-7/POSTN cells did not form any chondrocytic nodules under identical conditions.

**Figure 4:**
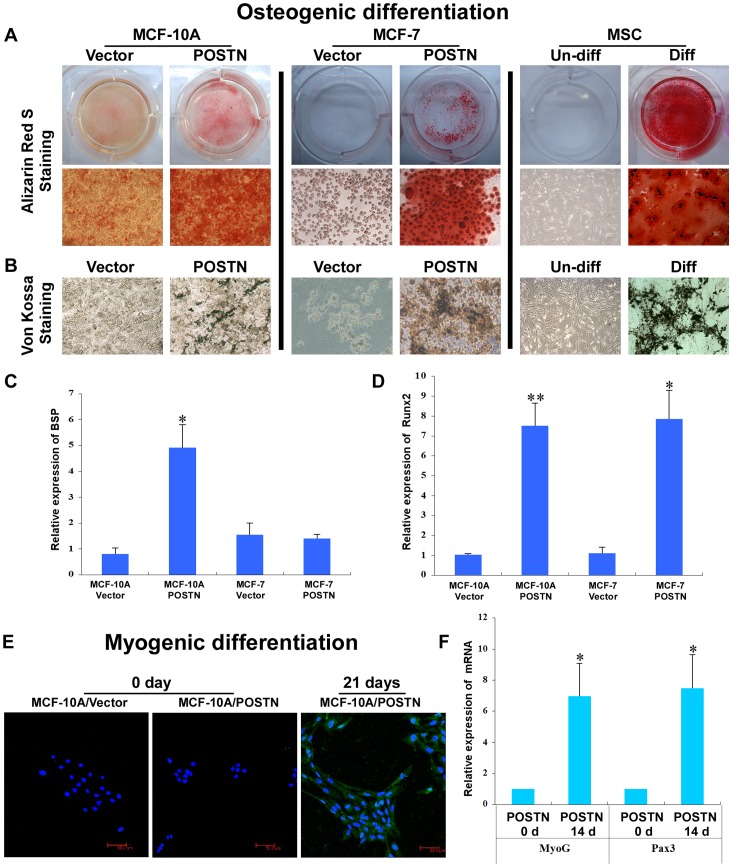
POSTN induces osteoblastic and myogenic differentiation. **A, B.** Following culture in osteoblastic differentiation media for 21 days, MCF-10A/POSTN, MCF-7/POSTN cells and hMSCs were positive for alizarin red S and von Kossa staining. **C, D.** Relative levels of mRNAs encoding BSP and Runx2 in MCF-10A and MCF-7 cells expressing the vector or POSTN were determined by real-time RT-PCR. *Cyclophilin G* mRNA was used to normalize the variability in template loading. The data are the means ± SD. *P<0.05, **P<0.01. **E.** Following myogenic differentiation for 4 weeks, MCF-10A/POSTN cells stained positive for CD56. MCF-7/POSTN cells died under the same myogenic differentiation condition and did not differentiate into myogenic lineages (data not shown). **F.** Real-time RT-PCR analysis of *MyoG* and *Pax3* showing the expression of myogenic markers. The data are the means ± SD. *P<0.05.

**Figure 5:**
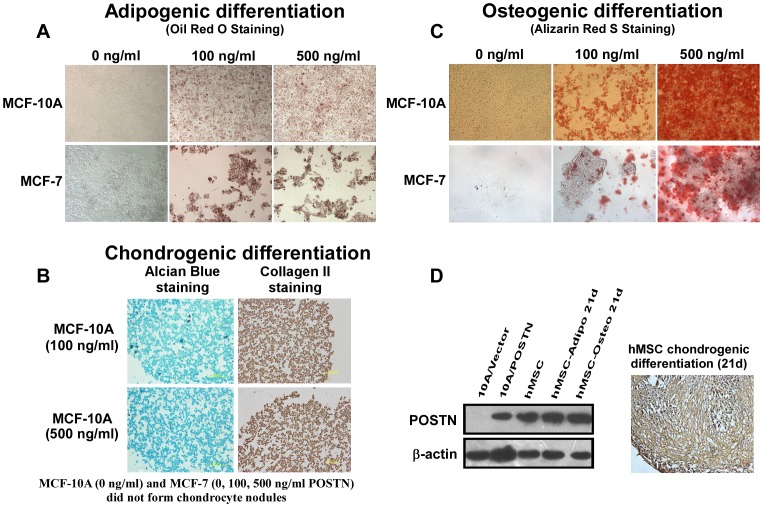
POSTN induces multilineage differentiation and is highly expressed in BM-MSCs and their derived cells *in vitro*. **A.** Following adipogenic differentiation, recombinant POSTN protein-treated MCF-10A and MCF-7 cells are positive for Oil Red O staining. **B.** Recombinant POSTN protein promoted the formation of chondrocytic nodules by MCF-10A cells. The differentiated cells are positive for alcian blue 8 GX staining (left panel). Immunohistochemistry was performed on chondrocyte sections using antibody against collagen II (right panel). MCF-7 cells did not form any chondrocytic nodules under identical conditions. **C.** Following culture in osteoblastic differentiation media for 21 days, recombinant POSTN-treated MCF-10A and MCF-7 cells are positive for Alizarin Red S staining. **D.** BM-MSCs and their derived cells expressed a high level of POSTN detected by western blotting or immunohistochemical analysis. Chondrocytic nodules formed by BM-MSCs were embedded by paraffin, cut into 5-µm-thick sections, then the sections were stained with monoclonal POSTN antibody.

To explore whether the mesenchymal-like cells induced by ectopic POSTN expression exhibit the multilineage differentiation potential of MSCs, we further characterised the MSC traits of MCF-10A/POSTN cells. We found that MCF-10A/POSTN cells exhibited the typical developmental potential of MSCs to differentiate into oil red O-positive and fluorescent LipidTox-positive adipocytes, alcian blue-positive chondrocytes, and alizarin red S-positive and von Kossa-positive mature osteoblasts when cultured in the appropriate differentiation conditions (Figure 3A, 3D, 4A, 4B). Real-time RT-PCR analysis showed that the adipocyte markers *PPARγ*and *ADFP* (Figure 3B, C) and the osteoblast markers *BSP* and *Runx2* (Figure 4C, D) are markedly upregulated in MCF-10A/POSTN cells grown under adipogenic or osteogenic differentiation conditions for 21 days, but not in MCF-10A/Vector cells. MCF-10A/POSTN cells can form chondrocytic nodules that are positive for collagen II, whereas MCF-10A/Vector cells did not form any chondrocyte nodules under identical conditions (Figure 3D). Moreover, MCF-10A/POSTN cells can differentiate into a CD56-positive myogenic lineage with increased expression of the myogenic markers *MyoG* and *Pax3* under myogenic differentiation culture for 4 weeks, but not the vector cells (Figure 4E, F). We further demonstrate that POSTN endows MCF-7 cells with the potential to differentiate into adipocytes and osteoblasts (Figure 3A, 4A, 4B), but not into chondrogenic and myogenic lineages (data not shown). Real-time RT-PCR analysis also showed that the adipocyte markers *ADFP* (Figure 3C) and the osteoblast markers *Runx2* (Figure 4D) are markedly upregulated in MCF-7/POSTN cells grown under adipogenic or osteogenic differentiation conditions for 21 days when compared with MCF-7/Vector control cells. We further confirmed these results by treating human mammary epithelial cells and BCCs with human recombinant POSTN protein (Figure 5A, B, C). These observations indicate that POSTN promotes MCF-10A and MCF-7 cells to exhibit multilineage differentiation potentials, in part, similar to MSCs.

### POSTN Promotes Tumor Growth and Enhances Breast Cancer Progression

**Figure 6:**
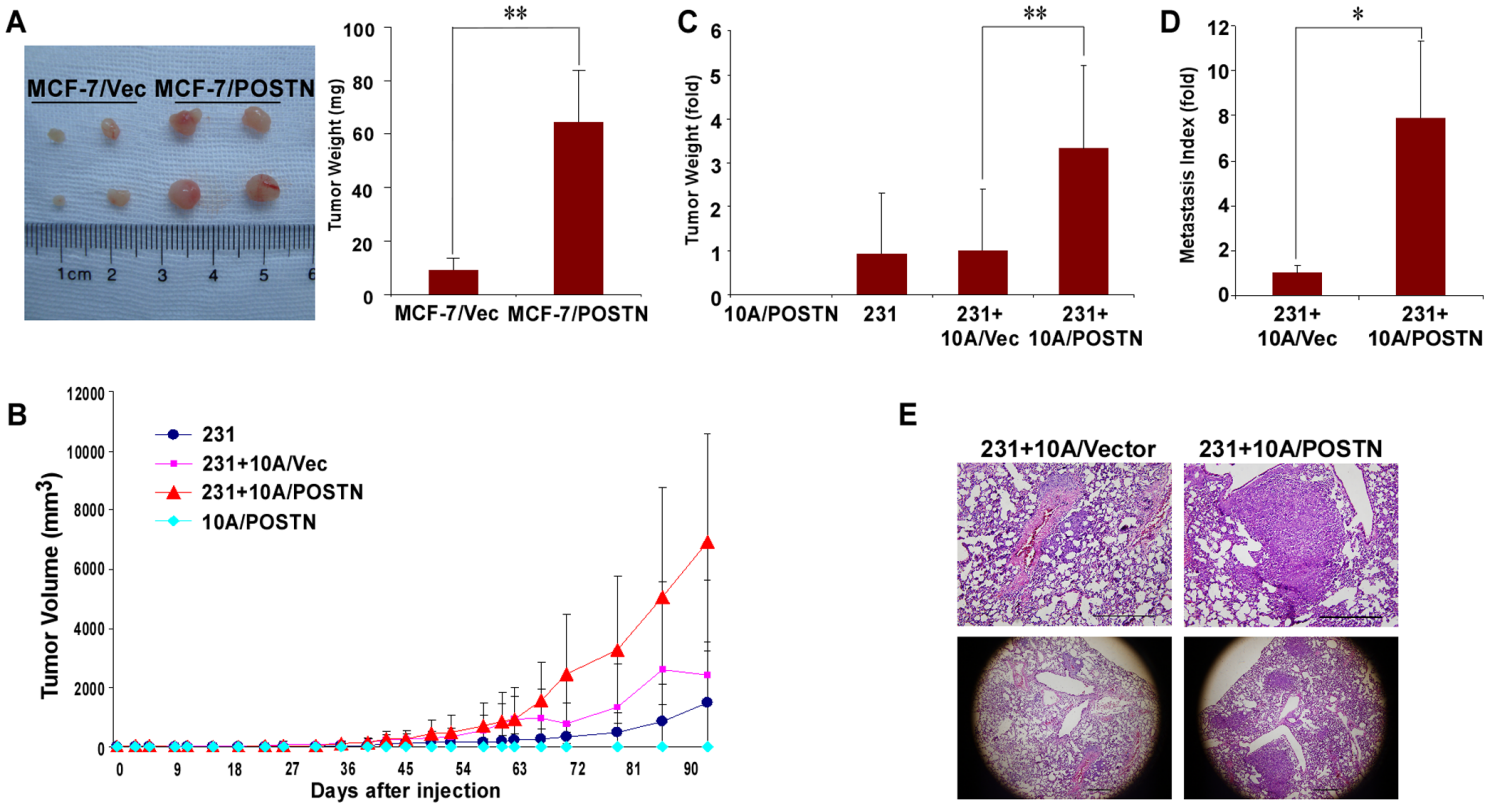
POSTN-overexpressing cells accelerates the tumor growth and metastatic properties of BCCs. **A.** 2.5×10^6^ MCF-7/Vector or MCF-7/POSTN cells were injected subcutaneously into 5- to 6-week-old female Balb/c nude mice (n = 4 mice per group). Mice were sacrificed 30 days after injection and examined for the growth of subcutaneous tumors. **P<0.01. **B.** Tumor weight measurements of 1×10^6^ MDA-MB-231 cells subcutaneously injected into nude mice with 3×10^6^ MCF-10A/Vector cells or MCF-10A/POSTN cells. **C.** The tumors were measured once or twice per week. Circles, MDA-MB-231 cells; squares, MDA-MB-231 cells plus MCF-10A/Vector cells; triangles, MDA-MB-231 cells plus MCF-10A/POSTN cells; diamonds, MCF-10A/POSTN cells (n = 4 mice per group). **P<0.01. **D.** Mice subcutaneously implanted with 3×10^6^ MDA-MB-231 mixed with 9×10^6^ MCF-10A/Vector cells or with MCF-10A/POSTN cells (n = 3 mice per group). Twelve weeks after cell injection, the mice were euthanized and the lung metastasis indices for each tumor bearing mouse were determined. *P<0.05. **E.** Representative haematoxylin-and-eosin-stained sections of lungs of mice bearing the indicated tumors in (D). Scale bar = 500 µm.

Interestingly, we also found that human BM-MSCs and their derived adipocytes, chondrocytes, and osteoblasts express high levels of POSTN *in vitro* (Figure 5D). BM-MSCs and their derived cells can integrate into the tumor-associated stroma and promote breast tumor growth and metastasis [26], [27]. These data indicate that POSTN might endow cancer cells with some of the phenotypic characteristics of BM-MSCs and that BM-MSCs and their derived cells might be another source of stromal POSTN in tumor microenvironment. To investigate whether POSTN promotes tumorigenesis of BCCs, we orthotopically implanted mice with MCF-7/Vector or MCF-7/POSTN cells. As shown in Figure 6A, the volume and weight of tumors in the mice bearing with MCF-7/POSTN cells after 30 days injection were marked increased compared to the MCF-7/Vector group (P<0.01). To further investigate whether POSTN-expressing cells also have the MSC-like potential to enhance tumor progression, we established an *in vivo* xenograft model of MDA-MB-231 cells subcutaneously implanted in Balb/c nude mice alone or mixed with MCF-10A vector cells or POSTN-overexpressing MCF-10A cells. We found that MCF-10A/POSTN cells did not result in tumor but enhanced the growth of MDA-MB-231 tumors. MCF-10A/POSTN cells augmented the volume and weight of MDA-MB-231 tumors compared to MCF-10A/Vector cells (P<0.01) (Figure 6B, C). However, implantation of MCF-10A/POSTN in nearby separate sites of MDA-MB-231 cells injection did not affect the growth of primary tumors (data not shown), indicating that MCF-10A/POSTN cells enhance tumor growth only when they interact directly with BCCs. Interestingly, mice bearing tumors resulting from implanted MDA-MB-231 cells mixed with MCF-10A/POSTN cells displayed a significant increase in the numbers of micro- and macrometastatic tumors in the lungs compared with MDA-MB-231 mixed with MCF-10A/Vector group (P<0.05) (Figure 6D, E). Taken together, POSTN-expressing MCF-10A cells have the ability to enhance breast tumor growth and metastasis.

## Discussion

Our results provide a new view for understanding the multifaceted roles of POSTN in breast cancer progression. CSCs and the EMT both contribute to tumor heterogeneity and tumor progression. As an ECM protein, POSTN has been implicated in the induction of the EMT during development, inflammation and tumor progression [14], [15], [28]. Fibroblast-derived POSTN was identified to be a limiting factor for lung metastasis of mouse breast tumors and is required for murine breast CSC maintenance. POSTN^−/−^ murine primary breast tumor cells fail to form tumorspheres, but this phenotype can be rescued by adding POSTN protein to primary cultures [23]. Interestingly, a recent report showed that POSTN is expressed higher in the sorted human CD44^+^/CD24^−/^line^−^ breast cancer stem cells compared to the control cells and the levels of POSTN are related to the CSC ratio in human breast cancer specimens [29]. Here, we demonstrate that the ectopic expression of POSTN endows human mammary epithelial cells and BCCs with stem cell-like or CSC-like capabilities and a mesenchymal phenotype. Therefore, POSTN may contribute to breast cancer progression via endowing normal and malignant cells with mesenchymal traits and stem cell-like properties.

BM-MSCs are characterised by their multipotent capacity to differentiate into many different cell types, such as osteoblasts, chondrocytes, and adipocytes, as well as other cells [30], [31]. MSCs can specifically migrate to and engraft at tumor sites and become a part of the tumor microenvironment, where they enhance the growth of the primary tumor and facilitate metastatic dissemination to distant organs [27], [32], [33], [34]. OPN, a matricellular protein, contributes to BMDC activation and the subsequent outgrowth of disseminated metastatic cells [33]. BM-MSCs can promote breast tumor growth by regulating CSC population via cytokine networks [35]. Interestingly, here we demonstrated that POSTN is highly expressed in human BM-MSCs and their derived adipocytes, osteoblasts and chondrocytes. Previous data have shown that POSTN is highly expressed in human mesenchymal stromal cells and their derived cells [36]. These data suggest that POSTN may play a critical role in modulating the mesenchymal state and the multipotent differentiation potentials of MSCs. We also observed similarities between POSTN-expressing human mammary epithelial cells and MSCs with respect to their multilineage differentiation as well as the ability to promote the metastatic potency of weakly metastatic human BCCs *in vivo*, indicating that POSTN-overexpressing human mammary epithelial cells exhibit multilineage differentiation potentials and pro-metastatic abilities similar to MSCs. Moreover, POSTN also induces some of the capacities of MSCs in human BCCs. These data demonstrate that POSTN-expressing human mammary epithelial cells and BCCs are multipotent and that MSCs and their derived cells might be another source of stromal POSTN in regulating the stemness of breast CSCs during the establishment of lung metastasis. Current data revealed that POSTN can directly interact with collagen I, fibronectin and Notch1 via its EMI domain and interact with tenascin C and BMP-1 via the FAS I domains. POSTN can recruit BMP-1 onto the fibronectin matrix to promote lysyl oxidase activity for collagen cross-linkage [10], [37]. These studies highlight the importance of POSTN in ECM homeostasis and in tissue microenvironment remodelling. Because the matrix elasticity can direct stem cell lineage specification of MSCs and MSCs can differentiate into different terminal differentiated cells under different biophysical conditions, POSTN might contribute to modulate the stemness and differentiation of MSCs by interacting with different ECM proteins to remodel the biophysical microenvironment and/or to regulate signalling in the cells.

**Figure 7:**
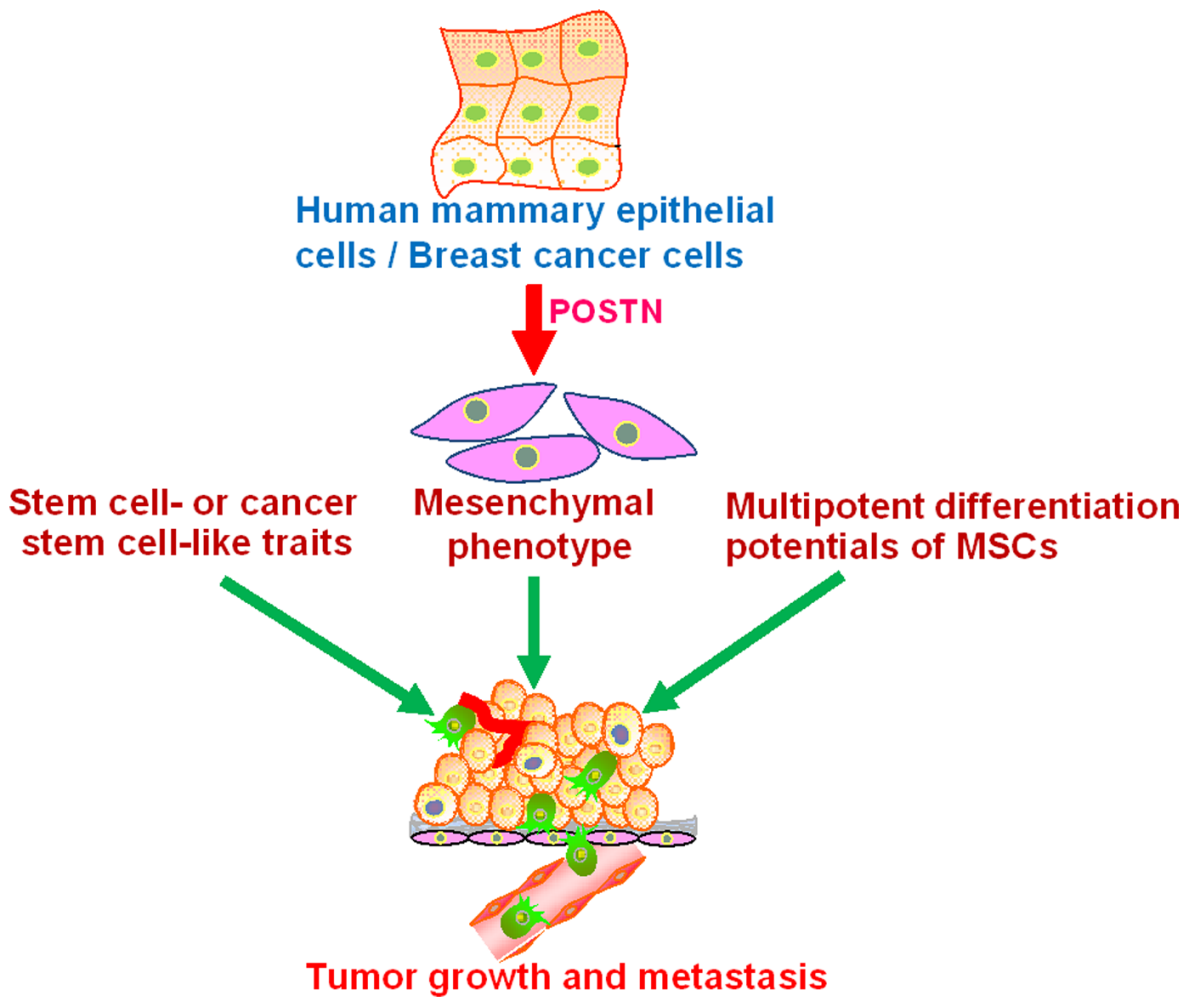
Model of role of POSTN in mammary epithelial neoplasia and metastasis. POSTN might confer mammary epithelial cells and BCCs with stem cell-like traits and a mesenchymal phenotype, as well as the multipotent potentials of MSCs to promote tumorigenesis and metastasis.

Interestingly, TGF-β, Twist1 and Snai1 can generate stem cells via EMT [25], and these EMT-derived human mammoray epithelial cells exhibit multilineage differentiation potentials similar to MSCs [30]. Stem-like cells from melanoma spheres can differentiate into melanocytic, adipocytic, osteoblastic, and chondrocytic lineages [38]. ΔNp63α can endow normal human keratinocytes with EMT traits and multipotent stem cell capacities [39]. Adult human retinal pigment epithelium cells can be activated into a multipotent stem cell that generates mesenchymal derivatives [40]. Moreover, glioblastoma stem cells were shown recently to differentiate into endothelial cells and pericytes that mediate tumor growth and metastasis [41], [42], [43]. The extracellular protease ADAMTS1 promotes some tumor cells to mimic an endothelial-like phenotype [44]. POSTN significantly promotes angiogenesis in human colon and breast cancers [17], [20]. Therefore, tumor stroma may contain the CSC-derived endothelial cells and the mesenchymally-transformed epithelial cancer cells as well as the stem-like cells with multilineage differentiation potentials induced by POSTN or other regulators, which participate with the physiologic stroma cells in the creation and maintenance of the tumor microenvironment and regulation of the stemness of CSCs. Epithelial cancer cells might acquire a mesenchymal phenotype and cancer stem cell-like traits, as well as the multipotent potential of MSCs to promote tumorigenesis and metastasis (Figure 7). Although it remains a debate as to whether the interconversion between epithelial and mesenchymal phenotypes is a general multipotent stem-like cell mechanism or is limited to rare oncogene sets, these data reveal the complexity of tumor heterogeneity and cell plasticity within tumor cell populations and their associated stromal compartments. Further understanding of the cellular and molecular mechanisms by which POSTN promotes metastasis and identification of POSTN functional modulators may hold promise for developing new strategies to inhibit tumor metastasis.

## Materials and Methods

### Ethics Statement

Animal studies were approved and performed according to the guidelines of the Animal Care and Use Committee of Xiamen University (Permit Number: XMULAC20120117-1).

### Cell Culture and Generation of Cell Lines

MCF-10A human mammary epithelial cells, MCF-7 and MDA-MB-231 breast cancer cells were provided by ATCC and maintained as described previously [45], [46]. Human bone marrow-derived MSCs (BM-MSCs) were provided by the Beijing Stem Cells Bank (Beijing, China) and maintained in MSC expansion medium (Millipore). Recombinant Human POSTN was purchased from R&D Systems. The human *POSTN* cDNA was provided by Professor Shideng Bao (Stem Cell Biology and Regenerative Medicine, Lerner Research Institute, Cleveland Clinic) and subcloned into a pBABE-puro expression vector. MCF-10A and MCF-7 cell lines that constitutively expressed either POSTN or empty vector were generated by retroviral infection as described previously [47].

### Western Blot and Immunostaining Assays

Western blot and immunostaining analyses were performed as described previously [47]. For the frozen section, chondrocyte nodules were embedded in OCT compound (Tissue-Tek) for sectioning and immunostaining analysis. The primary antibodies used included antibodies to POSTN (AdipoGen), vimentin (R&D System), E-cadherin, N-cadherin, fibronectin (BD Biosciences), α-SMA (Santa Cruz), collagen II (Abcam) and β-actin (Millipore).

### Real-time RT-PCR

RNA was extracted using TRIzol reagent (Invitrogen, Carlsbad, CA) and the expression levels of the mRNAs were determined by real-time RT-PCR using SYBR Green (Roche). *CyClophilin G* was used as an internal control. All data represent the average of three repeated experiments. The primer sequences are listed below:

*PPARγ*, forward: 5′-CTTGCAGTGGGGATGT-3′

*PPARγ*, reverse: 5′-CTTTGGTCAGCGGGAA-3′

*ADFP*, forward: 5′-CGCTGTCACTGGGGCAAAAGA-3′

*ADFP*, reverse: 5′-ATCCGACTCCCCAAGACTGTGTTA-3′

*BSP*, forward: 5′-CGGAGGAGACAATGGAGAAG-3′

*BSP*, reverse: 5′-GACGCCCGTGTATTCGTACT-3′

*RUNX2*, forward: 5′-ACAGTAGATGGACCTCGGGA-3′

*RUNX2*, reverse: 5′-ATACTGGGATGAGGAATGCG-3′

*MyoG*, forward: 5′-GCCAGACTATCCCCTTCCTC-3′

*MyoG*, reverse: 5′-GAGGCCGCGTTATGATAAAA-3′

*Pax3*, forward: 5′-GGAGAAGAGGAAGACCTGGAGCAATAAA-3′

*Pax3*, reverse: 5′-GCACGCACACAAGCAAATGGAA-3′

*Cyclophilin G*, forward: 5′-CTTGTCAATGGCCAACAGAGG-3′

*Cyclophilin G*, reverse: 5′-GCCCATCTAAATGAGGAGTTGGT-3′

### Flow Cytometry

The identification of CD44^high^/CD24^low^ cells was performed using monoclonal anti-CD44-FITC (clone G44-26) and anti-CD24-PE (clone ML5) antibodies (BD Bioscience). FITC Mouse IgG2b K Isotype Control and PE Mouse IgG2a K Isotype Control (BD Biosciences) were used as isotype controls. Cells were labelled and CD44/CD24 markers were analysed using a FACSCalibur flow cytometer (BD Biosciences) as described previously [47].

### Mammosphere Culture

Mammosphere culture was performed as described previously with a slight modification [48]. To induce sphere formation, cells were dissociated to single cells by 0.05% trypsin-EDTA solution and plated into 24-well ultra-low attachment plates at a density of 4000 viable cells/ml. Cells were grown in a serum-free DMEM/F12 medium, supplemented with B27 (Invitrogen), 20 ng/ml EGF, 20 ng/ml bFGF, 4 µg/ml Heparin (Sigma), and 1% methyl cellulose. The mammospheres were cultured for 7 days.

### Matrigel-coated Transwell Invasion Assay

Cell invasion was determined using Transwell plates (Corning) with a pore size of 8 µm. In total, 2×10^5^ cells were seeded in serum-free medium in the upper chambers (the Matrigel is on the upper surface of the chambers, and fibronectin is on the bottom surface of the chambers), while complete media were added to the bottom chambers. After 48 h, the cells on the upper surface of the filters were carefully removed with a cotton swab and the cells that had traversed to the verse face of the membrane were fixed and stained with crystal violet.

### Osteoblast Differentiation

3×10^4^ MCF-10A or MCF-7 cells expressing the empty vector or POSTN as well as BM-MSCs were cultured in OsteoDiff media containing 10 mmol/l β-glycerol-2-phosphate, 50 mg/l ascorbic acid and 0.1 µmol/l dexamethasone. The osteoblast differentiation assay was performed as described previously [30].

### Adipocyte Differentiation

6×10^4^ cells were cultured in AdipoDiff medium containing 1 µmol/l dexamethasone, 10 mg/l insulin, 0.5 mmol/l IBMX and 100 µmol/l indomethacin in a six-well cell culture dish. The adipocyte differentiation assay was performed as described previously [30].

### Chondrogenic Differentiation

4×10^5^ cells were incubated in ChondroDiff medium containing L-DMEM, 1% penicillin-streptomycin, 0.1 µmol/l dexamethasone, 1 mmol/l sodium pyruvate, 10 ng/ml TGF-β1, 50 mg/l ascorbic acid, 6.25 µg/ml insulin, and 6.25 µg/ml transferrin. The chondrogenic differentiation assay was performed as described previously [30].

### Myogenic Differentiation

6×10^4^ cells were cultured in MyoDiff medium for myogenic differentiation as described previously [38].

### Animal Studies

To measure tumourigenicity *in vivo* in nude mice, 2.5×10^6^ MCF-7/Vector or MCF-7/POSTN cells were mixed with an equal volume of Matrigel (BD Biosciences) and injected subcutaneously into 5- to 6-week-old female Balb/c nude mice (n = 4 mice per group). Mice were treated weekly with 20 µl of a 10^−2^ M ethanolic solution of E2 applied to the neck skin. Mice were sacrificed 30 days after injection and examined for the growth of subcutaneous tumors. 1×10^6^ MDA-MB-231 cells alone or mixed with 3×10^6^ MCF-10A/Vector cells or MCF-10A/POSTN cells were subcutaneously injected into 5- to 6-week-old female Balb/c nude mice (n = 4 mice per group). The tumors were measured 1–2 times per week and calculated as 0.5×length×width^2^. The tumors were weighted at the end of the experiments. For the lung metastasis assay, 5- to 6-week-old female Balb/c nude mice were implanted subcutaneously with 3×10^6^ MDA-MB-231 mixed with 9×10^6^ MCF-10A vector cells or with MCF-10A/POSTN cells (n = 3 mice per group). Three months after the tumor cell injection, the mice were euthanized and examined for metastatic tumors in the lungs. The lung metastasis index for each mouse was calculated as the ratio of the number of colonies observed in serial sections of each lung divided by the mass of the primary tumor (in grams).

### Statistical Analysis

The quantitative data are presented as the means ± s.d. and were analysed by the Student’s *t*-test. A level of P<0.05 was considered statistically significant.

## Supporting Information

Figure S1 **POSTN overexpression in non-stem MCF-10A and MCF-7 cells enhances CD44^high^/CD24^low^ subpopulations. A, B.** The sorted main non-stem cell subpopulation in MCF-10A (CD44^high^/CD24^high^) and MCF-7 (CD44^low^/CD24^high^) expressed either POSTN or empty vectors. **C, D.** The percentages of CD44^high^/CD24^low^ subpopulations in non-stem MCF-10A/Vector and MCF-7/Vector cells and their POSTN-overexpressing cells. The data are the means ± SD. **P<0.01.
(TIF)
