# Supplementary figures and images for: Periostin Contributes to the Acquisition of Multipotent Stem Cell-Like Properties in Human Mammary Epithelial Cells and Breast Cancer Cells
Source: PLoS One. 2013 Aug 29;8(8):e72962. doi: 10.1371/journal.pone.0072962 (PMC3756944; doi:10.1371/journal.pone.0072962)

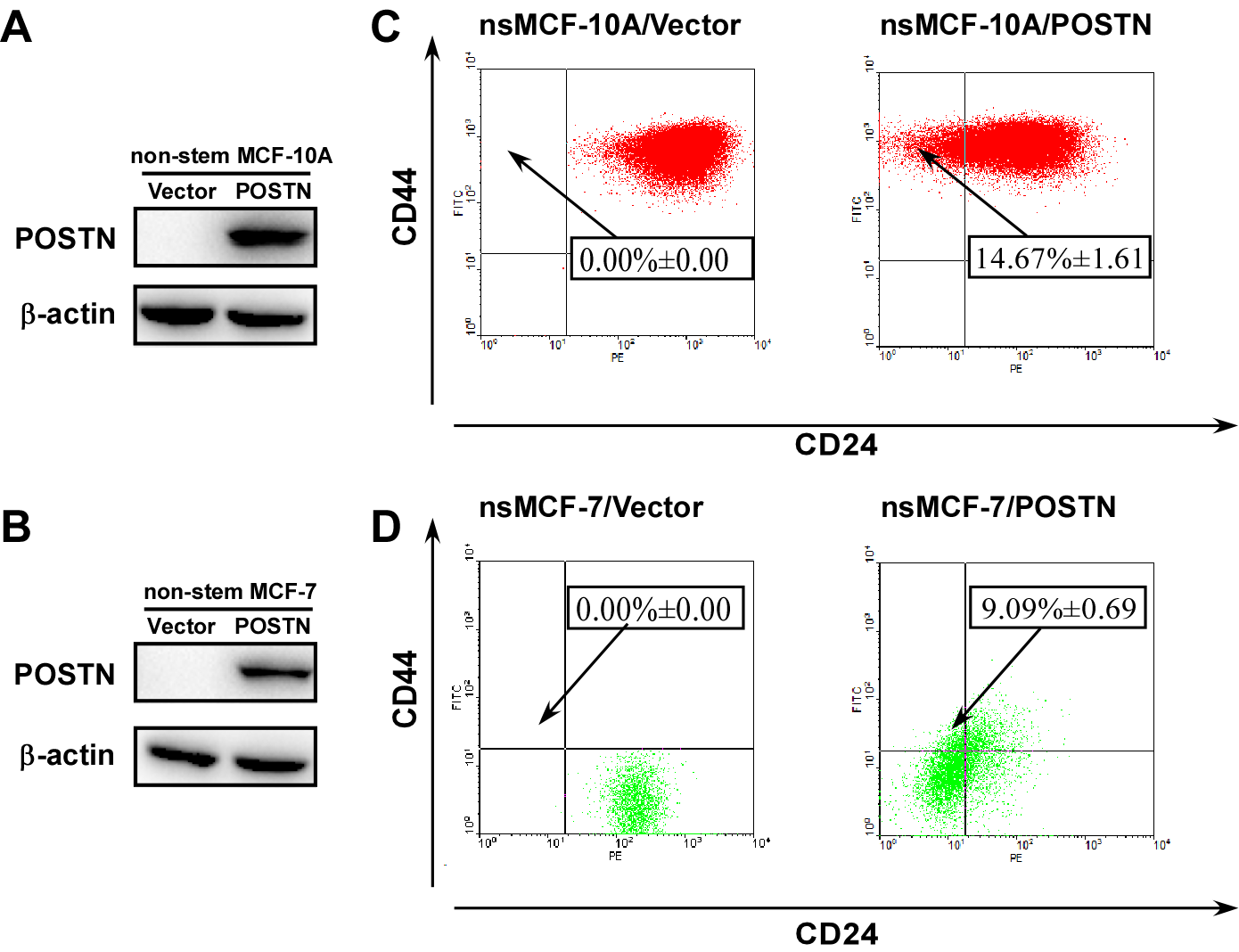

Supplement: Figure S1 — POSTN overexpression in non-stem MCF-10A and MCF-7 cells enhances CD44high/CD24low subpopulations. A, B. The sorted main non-stem cell subpopulation in MCF-10A (CD44high/CD24high) and MCF-7 (CD44low/CD24high) expressed either POSTN or empty vectors. C, D. The percentages of CD44high/CD24low subpopulations in non-stem MCF-10A/Vector and MCF-7/Vector cells and their POSTN-overexpressing cells. The data are the means ± SD. **P<0.01. (TIF) [file pone.0072962.s001.tif]
